# miRNA-19b-3p Stimulates Cardiomyocyte Apoptosis Induced by Myocardial Ischemia Reperfusion via Downregulating PTEN

**DOI:** 10.1155/2021/9956666

**Published:** 2021-12-16

**Authors:** Ke Li, Xujie Ya, Xiujuan Duan, Yang Li, Xuefeng Lin

**Affiliations:** ^1^Department of Cardiology, Nanyang Second People's Hospital, Nanyang, China; ^2^The First Department of Cardiology, The Eighth People's Hospital of Hengshui City, Hengshui, China; ^3^The Second Department of Cardiology, The Eighth People's Hospital of Hengshui City, Hengshui, China; ^4^Department of Cardiovascular Medicine, The First Affiliated Hospital of Baotou Mdical College, Baotou, China

## Abstract

**Objective:**

To clarify the function of miRNA-19b-3p in accelerating myocardial ischemia-reperfusion injury- (MIRI-) induced cardiomyocyte apoptosis by downregulating gene of phosphate and tension homology deleted on chromsome ten (PTEN), thus influencing the progression of acute myocardial infarction.

**Materials and Methods:**

miRNA-19b-3p and PTEN levels in HCM cells undergoing hypoxia/reoxygenation (H/R) were determined. Meanwhile, activities of myocardium injury markers [lactate dehydrogenase (LDH), malondialdehyde; malonic dialdehyde (MDA), superoxide dismutase (SOD), and glutathione peroxidase (GSH-PX)] in H/R-induced HCM cells were tested. Through dual-luciferase reporter gene assay, the binding between miRNA-19b-3p and PTEN was verified. Regulatory effects of miRNA-19b-3p and PTEN on apoptotic rate and apoptosis-associated gene expressions (proapoptotic protein Bcl-2 associated X protein (Bax), antiapoptotic protein B-cell lymphoma-2 (Bcl-2), and cytochrome C) in H/R-induced human cardiac myocytes (HCM) cells were examined.

**Results:**

miRNA-19b-3p was upregulated, while PTEN was downregulated in H/R-induced HCM cells. Knockdown of miRNA-19b-3p decreased activities of LDH, MDA, and GSH-PX, but increased SOD level in H/R-induced HCM cells. The binding between miRNA-19b-3p and PTEN was confirmed. More importantly, knockdown of miRNA-19b-3p reduced apoptotic rate, downregulated proapoptosis gene expressions (Bax and cytochrome C), and upregulated antiapoptosis gene expression (Bcl-2), which were reversed by silence of PTEN.

**Conclusions:**

miRNA-19b-3p is upregulated in HCM cells undergoing hypoxia and reoxygenation, which accelerates MIRI-induced cardiomyocyte apoptosis through downregulating PTEN.

## 1. Introduction

Acute myocardial infarction (AMI) is an important cause of death and disability worldwide [1–3]. Timely myocardial reperfusion is the most effective intervention for alleviating ischemia-induced myocardium injury. However, reperfusion itself induces myocardial cell death, that is, myocardial ischemia-reperfusion injury (MIRI) [4–6]. Apoptosis is a process of programmed cell death influencing MIRI and cardiomyocyte loss during cardiac remodeling at post-AMI [7]. A growing number of evidences have suggested that cardiomyocyte apoptosis occurs primarily in the surviving myocardium following ischemia [8]. It is necessary to uncover the pathogenic mechanism of MIRI and develop effective therapeutic targets for clinical treatment of AMI.

miRNAs are a class of noncoding DNAs expressed in eukaryotic cells, ranging in length from 20 to 25 nucleotides [9–11]. Mature miRNAs are processed by primary transcripts through various nucleases, which are then assembled into an RNA-induced silencing complex (RISC). Subsequently, RISC binds 3′UTR of target mRNAs through complementary base pairing, thus degrading mRNAs or inhibiting their translation [12, 13]. miRNAs are extensively distributed in different types of cells and human diseases, such as ischemic cardiomyopathy [14], cardiac remodeling [15], heart failure [16], and arrhythmia [17]. In recent years, critical functions of miRNAs in MIRI have been identified [18–20]. These miRNAs could be utilized as therapeutic targets for clinical treatment of AMI.

miRNA-19b-3p is a member of the miR-17-92 cluster located on the human chromatin 13. Biological functions of miRNA-19b-3p have been discovered in multiple types of tumors [21–25]. In a recent study, exosomal miRNA-19b-3p of tubular epithelial cells promotes M1 macrophage activation in kidney injury [26]. Further, circulating miR-19a-3p and miR-19b-3p characterize the human aging process and their isomiRs associate with healthy status at extreme ages [27]. However, the role of miR-19b-3p in AMI was unknown. In this paper, regulatory effects of miRNA-19b-3p on AMI-induced cardiomyocyte apoptosis were determined.

## 2. Materials and Methods

### 2.1. Cell Culture and H/R Induction

Human cardiac myocytes (HCM) provided by American Type Culture Collection (ATCC, Manassas, VA, USA) were cultured in Dulbecco's Modified Eagle's Medium (DMEM, Sigma, Louis, MO, USA) containing 10% fetal bovine serum (FBS, Invitrogen, Carlsbad, CA, USA). HCM cells were cultured in a humidified incubator containing 5% CO_2_ and 95% N_2_ for 4 h. Later, reoxygenation was conducted by cell culture in DMEM containing 10% glycerol in a humidified incubator containing 5% CO_2_ and 95% air for 3 h. After normoxic culture overnight, cells were harvested for functional experiments. Normoxic-preconditioning HCM cells were harvested as controls.

### 2.2. Transfection

Transfection vectors were provided by GeneChem, (Shanghai, China). Cell transfection was conducted using Lipofectamine TM 2000 (Invitrogen, Carlsbad, CA, USA). Six hours later, transfection efficacy was verified. Transfected cells were collected for H/R exposure.

### 2.3. Real-Time Quantitative Polymerase Chain Reaction (RT-qPCR)

TRIzol Reagent (Invitrogen, Carlsbad, CA, USA) was applied for isolating cellular RNA. Complementary deoxyribonucleic acids (cDNAs) was obtained by reverse transcription of 2 *μ*g RNA using cDNA synthesis kit (TaKaRa, Tokyo, Japan) and amplified on the MiniOpticon qPCR determination system (Bio-Rad, Hercules, CA, USA). Relative level was calculated using the 2^-*ΔΔ*CT^ method. miRNA-19b-3p, F: 5′-AGUUUUGCAUGGAUUUGCAC-3′ and R: 5′-UUUGCAUGGAUUUGCACAUU-3′; PTEN, F: 5′-TGGTGAGGTTTGATCCGCATA-3′ and R: 5′-CCCAGTCAGAGGCGCTATG-3′; Bax, F: 5′-CACAACTCAGCGCAAACATT-3′ and R: 5′-ACAGCCATCTCTCTCCATGC-3′; Bcl-2, F: 5′-GAAGCACAGATGGTTGATGG-3′ and R: 5′-CAGCCTCACAAGGTTCCAAT-3′; cytochrome C, F: 5′-TAAATATGAGGGTGTCGC-3′ and R: 5′-AAGAATAGTTCCGTCCTG-3′.

### 2.4. Western Blot

Radio immunoprecipitation assay (RIPA) was applied for isolating cellular protein. Protein sample was quantified by bicinchoninic acid (BCA) method and underwent electrophoresis (Beyotime, Shanghai, China). Protein was transferred on a polyvinylidene fluoride (PVDF) membranes (Roche, Basel, Switzerland) and blocked in phosphate buffer saline (PBS) containing 5% skim milk for 2 h. Subsequently, membranes were reacted with primary antibodies at 4°C overnight and secondary antibodies for 2 h. Band exposure was achieved by enhanced chemiluminescence (ECL) and analyzed by Image Software (NIH, Bethesda, MD, USA).

### 2.5. Dual-Luciferase Reporter Gene Assay

Wild-type PTEN (NM_000314.8) 3′untranslated region (3′UTR) was amplified and inserted into the pGL3 vector. Predicted binding sequences between PTEN 3′UTR and miRNA-19b-3p were mutated using the QuickChange Site-Directed Mutagenesis Kit (Stratagene, Heidelberg, Germany). After cotransfection with miRNA-19b-3p mimics/NC and WT-PTEN/MT-PTEN for 48 h, relative luciferase activity was determined.

### 2.6. Cell Counting Kit-8 (CCK-8)

Cells were inoculated in a 96-well plate at 80% confluence. Viability was determined at the appointed time points using CCK-8 kit (Dojindo Laboratories, Kumamoto, Japan). Absorbance at 450 nm was recorded for plotting the viability curve.

### 2.7. Determination of Levels of LDH, MDA, SOD, and GSH-PX

Relative commercial kits were obtained from Sangon Biotech, (Shandong, China). Transfected cells were harvested for determining levels of myocardial injury markers based on the manufacturer's recommendations.

### 2.8. Flow Cytometry

Cells were digested in 0.25% trypsin, centrifuged, and washed with PBS for three times. Cells were dual-stained with Annexin-V-FITC (fluorescein isothiocyanate) and subjected to flow cytometry (FACSCalibur; BD Biosciences, Detroit, MI, USA) for measuring apoptotic rate.

### 2.9. Statistical Analysis

Statistical Product and Service Solutions (SPSS) 20.0 (SPSS, Chicago, IL, USA) was used for data analyses. Data were expressed as mean ± standard deviation. The Student *t*-test was applied for analyzing differences between the two groups.*p* < 0.05 was considered statistically significant.

## 3. Results

### 3.1. miRNA-19b-3p Was Upregulated in H/R-Induced Cells

**Figure 1:**
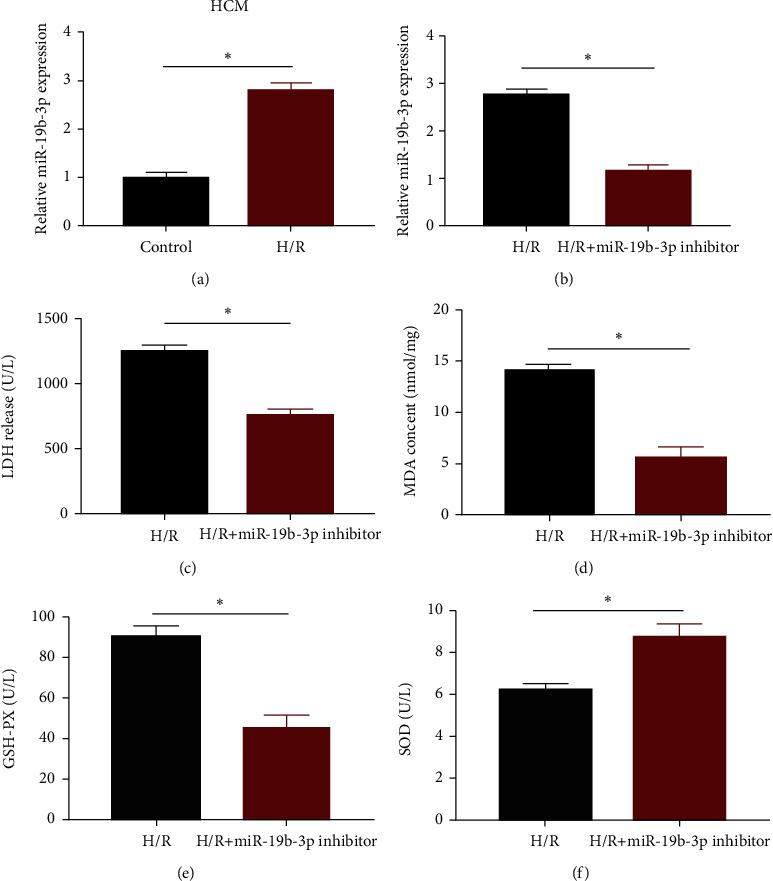
miRNA-19b-3p was upregulated in H/R-induced cells. (a) miRNA-19b-3p level in HCM cells undergoing normoxic or H/R induction. (b) Transfection efficacy of miRNA-19b-3p inhibitor. (c)–(f) Activities of LDH (c), MDA (d), GSH-PX (e), and SOD (f) in H/R-induced HCM cells or those transfected with miRNA-19b-3p inhibitor.

H/R was conducted in HCM cells to mimic the in vitro environment of MIRI. Compared with HCM cells under normoxic conditions, H/R induction markedly upregulated miRNA-19b-3p in HCM cells (Figure 1(a)). Subsequently, transfection of miRNA-19b-3p inhibitor markedly downregulated miRNA-19b-3p level in H/R-induced HCM cells, presenting an effective transfection efficacy (Figure 1(b)). Myocardial injury markers were determined here. As the data revealed, knockdown of miRNA-19b-3p decreased activities of LDH, MDA, and GSH-PX, but increased SOD level in H/R-induced HCM cells (Figures 1(c)–1(f)). It is demonstrated that miRNA-19b-3p was involved in MIRI.

### 3.2. Knockdown of miRNA-19b-3p Alleviated Cardiomyocyte Apoptosis

**Figure 2:**
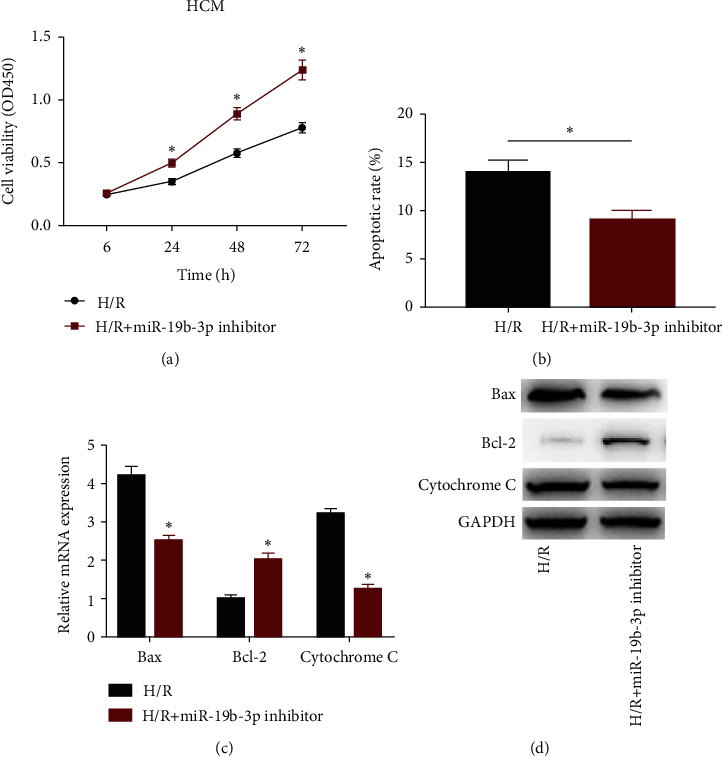
Knockdown of miRNA-19b-3p alleviated cardiomyocyte apoptosis. HCM cells were cultured in H/R environment and transfected either with miRNA-19b-3p inhibitor or not. (a) Viability. (b) Apoptotic rate. (c) The mRNA levels of Bax, Bcl-2, and cytochrome C. (d) The protein levels of Bax, Bcl-2, and cytochrome C.

In H/R-induced HCM cells, transfection of miRNA-19b-3p inhibitor accelerated cell viability (Figure 2(a)). Nevertheless, apoptotic rate decreased after knockdown of miRNA-19b-3p in H/R-induced HCM cells (Figure 2(b)). Expression levels of apoptosis-associated genes, Bax, Bcl-2, and cytochrome C were determined. Both mRNA and protein levels of Bax and cytochrome C were downregulated, and Bcl-2 was upregulated in H/R-induced HCM cells transfected with miRNA-19b-3p inhibitor (Figures 2(c) and 2(d)).

### 3.3. PTEN Was the Direct Target of miRNA-19b-3p

**Figure 3:**
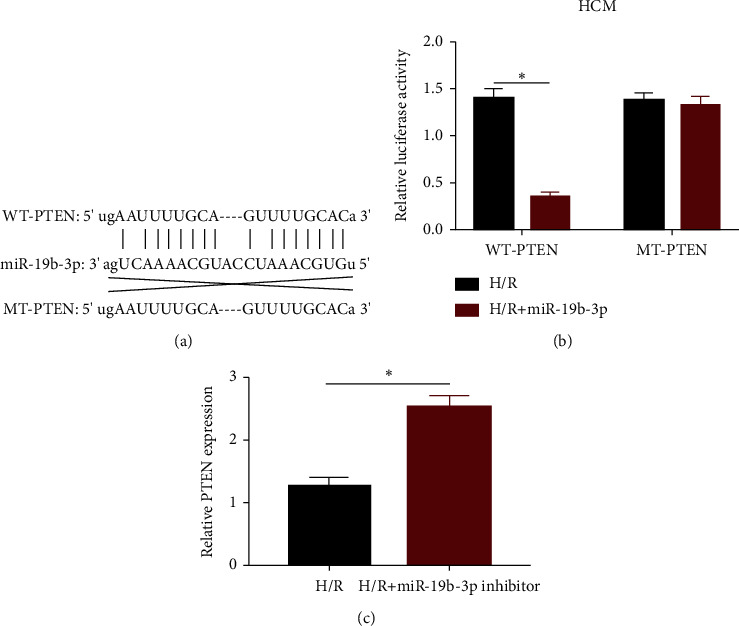
PTEN was the direct target of miRNA-19b-3p. (a) Potential binding sequences in the promoter regions of PTEN and miRNA-19b-3p. (b) Luciferase activity in HCM cells cotransfected with miRNA-19b-3p mimics/NC and WT-PTEN/MT-PTEN. (c) PTEN expression in H/R-induced HCM cells or those transfected with miRNA-19b-3p inhibitor.

To further uncover the mechanism of miRNA-19b-3p in influencing MIRI, we found potential binding sequences in the promoter regions of miRNA-19b-3p and PTEN as predicted in Targetscan (Figure 3(a)). Dual-luciferase reporter gene assay demonstrated that the overexpression of miRNA-19b-3p quenched luciferase activity in wild-type PTEN vector, while it did not affect mutant-type PTEN vector (Figure 3(b)). In addition, PTEN level was markedly upregulated in H/R-induced HCM cells transfected with miRNA-19b-3p inhibitor (Figure 3(c)). It is concluded that PTEN was the direct target of miRNA-19b-3p and negatively regulated by it.

### 3.4. miRNA-19b-3p Accelerated Cardiomyocyte Apoptosis by Downregulating PTEN

**Figure 4:**
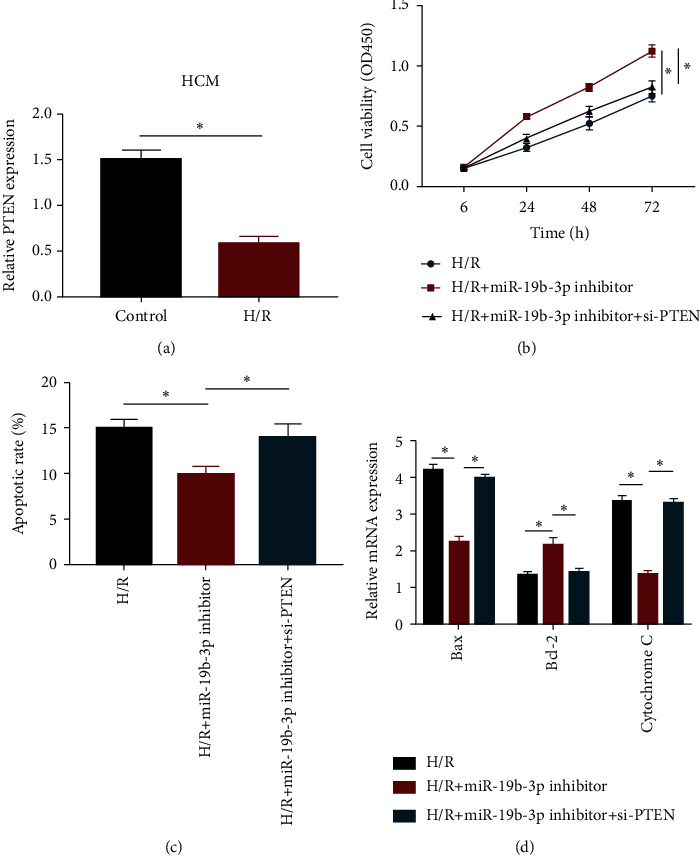
miRNA-19b-3p accelerated cardiomyocyte apoptosis by downregulating PTEN. (a) PTEN level in HCM cells undergoing normoxic or H/R induction. HCM cells were cultured in H/R environment and transfected with miRNA-19b-3p inhibitor or miRNA-19b-3p inhibitor + si-PTEN. (b) Viability. (c) Apoptotic rate. (d) The mRNA levels of Bax, Bcl-2, and cytochrome C.

Compared with HCM cells cultured in the normoxic environment, PTEN was markedly downregulated in H/R-induced cells (Figure 4(a)). It is speculated that PTEN was involved in cardiomyocyte apoptosis influenced by miRNA-19b-3p. CCK-8 assay showed that the enhanced viability in H/R-induced HCM cells with miRNA-19b-3p knockdown was partially reversed by cotransfection of si-PTEN (Figure 4(b)). Besides, decreased apoptotic rate after knockdown of miRNA-19b-3p was elevated by transfection of si-PTEN (Figure 4(c)). Similarly, regulatory effects of miRNA-19b-3p on apoptosis-associated gene expressions were reversed by silence of PTEN (Figure 4(d)). Therefore, PTEN was responsible for miRNA-19b-3p-mediated cardiomyocyte apoptosis following MIRI.

## 4. Discussion

Currently, thrombolysis, bypass surgery, and other interventions have been applied for reperfusion of blood flow and protection of ischemic myocardium [28]. Nevertheless, the sudden reperfusion of blood flow would result in secondary cardiovascular injury, that is, MIRI. MIRI results in cardiomyocyte apoptosis and necrosis, and even cardiac arrest [29]. Cell apoptosis is a vital event during the prognosis of MI. Inhibition of cardiomyocyte apoptosis and reduction of infarcted myocardium area could effectively alleviate the prognosis of AMI [30, 31].

Accumulating evidences have uncovered the role of miRNAs in regulating reperfusion in ischemic myocardium. In this paper, miRNA-19b-3p was upregulated in HCM cells under H/R precondition. Silence of miRNA-19b-3p markedly reduced activities of LDH, MDA, and GSH-PX and elevated SOD level in H/R-induced HCM cells. In addition, knockdown of miRNA-19b-3p resulted in viability elevation and apoptosis suppression in HCM cells. Our findings demonstrated the involvement of miRNA-19b-3p in MIRI-induced pathological changes. Furthermore, apoptosis-related genes were determined in H/R-induced HCM cells. Previous studies proposed that Bcl-2/Bax ratio is the key indicator reflecting the apoptotic level [32, 33]. Bcl-2 protein locates in the outer mitochondrial membrane, exerting an antiapoptosis function. Under normal circumstance, Bax is expressed in the cytoplasm. Once AMI occurs, apoptosis-related signaling triggers the translocation of cytoplasmic Bax into mitochondria, thus initiating the endogenous apoptosis. Here, silence of miRNA-19b-3p downregulated mRNA and protein levels of Bax and cytochrome C, and upregulated Bcl-2.

PTEN is a lipoprotein phosphatase that negatively regulates the PI3K/Akt pathway through PIP3 dephosphorylation and Akt translocation on the cell membrane [34, 35]. A recent study illustrated the crucial role of PTEN in mitochondrial-dependent apoptosis [36].

PTEN is considered to be an important pathway involved in MI. Previous studies have demonstrated that the upregulated miR-21 during MI affects collagen production by interfering with VEGF-mediated PTEN pathway [37]. In previous studies, miRNAs were reported to regulating target genes by binding to the 3′UTR area as a sponge thus to inhibit the translation of mRNA [38, 39]. In this paper, PTEN was confirmed to be the direct target of miRNA-19b-3p and negatively regulated by it. Besides, PTEN was lowly expressed in H/R-induced HCM cells. To elucidate the involvement of PTEN in HCM cell behaviors influenced by miRNA-19b-3p, gain-of-function experiments were conducted. Notably, knockdown of PTEN reversed regulatory effects of miRNA-19b-3p on apoptotic rate and apoptosis-associated gene expressions in H/R-induced HCM cells. As a result, PTEN was responsible for miRNA-19b-3p to influence MIRI-induced cardiomyocyte apoptosis.

## 5. Conclusions

miRNA-19b-3p is upregulated in HCM cells undergoing hypoxia and reoxygenation, which accelerates cardiomyocyte apoptosis through downregulating PTEN.

## Data Availability

The datasets used and analyzed during the current study are available from the corresponding author on reasonable request.
